# Eight-lncRNA signature of cervical cancer were identified by integrating DNA methylation, copy number variation and transcriptome data

**DOI:** 10.1186/s12967-021-02705-9

**Published:** 2021-02-08

**Authors:** Qihang Zhong, Minzhen Lu, Wanqiong Yuan, Yueyi Cui, Hanqiang Ouyang, Yong Fan, Zhaohui Wang, Congying Wu, Jie Qiao, Jing Hang

**Affiliations:** 1 Institute of Systems Biomedicine, School of Basic Medical Sciences, Peking University Health Science Center, Peking University, HaiDian District, No. 38 XueYuan Road, Beijing, 100191 China; 2 Center for Reproductive Medicine, Department of Obstetrics and Gynecology, Peking University Third Hospital, HaiDian District, No. 49 North HuaYuan Road, Beijing, 100191 China; 3 National Clinical Research Center for Obstetrics and Gynecology, Beijing, 100191 China; 4 Key Laboratory of Assisted Reproduction, Ministry of Education, Beijing, 100191 China; 5 Beijing Key Laboratory of Reproductive Endocrinology and Assisted Reproductive Technology, Beijing, 100191 China; 6 Department of Orthopedics, Peking University Third Hospital, Beijing, 100091 China; 7 Beijing Key Laboratory of Spinal Disease Research, Beijing, 100191 China; 8 Key Laboratory for Major Obstetric Diseases of Guangdong Province, The Third Affiliated Hospital of Guangzhou Medical University, Guangzhou, 510150 China; 9 State Key Laboratory of Molecular Developmental Biology, Institute of Genetics and Developmental Biology, Chinese Academy of Sciences, Beijing, China

**Keywords:** Copy number variation, Multi-omics integration analysis, lncRNA signature, Cervical cancer

## Abstract

**Background:**

Copy number variation (CNV) suggests genetic changes in malignant tumors. Abnormal expressions of long non-coding RNAs (lncRNAs) resulted from genomic and epigenetic abnormalities play a driving role in tumorigenesis of cervical cancer. However, the role of lncRNAs-related CNV in cervical cancer remained largely unclear.

**Methods:**

The data of messenger RNAs (mRNAs), DNA methylation, and DNA copy number were collected from 292 cervical cancer specimens. The prognosis-related subtypes of cervical cancer were determined by multi-omics integration analysis, and protein-coding genes (PCGs) and lncRNAs with subtype-specific expressions were identified. The CNV pattern of the subtype-specific lncRNAs was analyzed to identify the subtype-specific lncRNAs. A prognostic risk model based on lncRNAs was established by least absolute shrinkage and selection operator (LASSO).

**Results:**

Multi-omics integration analysis identified three molecular subtypes incorporating 617 differentially expressed lncRNAs and 1395 differentially expressed PCGs. The 617 lncRNAs were found to intersect with disease-related lncRNAs. Functional enrichment showed that 617 lncRNAs were mainly involved in tumor metabolism, immunity and other pathways, such as p53 and cAMP signaling pathways, which are closely related to the development of cervical cancer. Finally, according to CNV pattern consistent with differential expression analysis, we established a lncRNAs-based signature consisted of 8 lncRNAs, namely, RUSC1-AS1, LINC01990, LINC01411, LINC02099, H19, LINC00452, ADPGK-AS1, C1QTNF1-AS1. The interaction of the 8 lncRNAs showed a significantly poor prognosis of cervical cancer patients, which has also been verified in an independent dataset.

**Conclusion:**

Our study expanded the network of CNVs and improved the understanding on the regulatory network of lncRNAs in cervical cancer, providing novel biomarkers for the prognosis management of cervical cancer patients.

## Background

Cervical cancer is one of the most frequently diagnosed cancers and a leading cause of cancer deaths to women [1]. Annually there are 500,000 newly diagnosed cases of cervical cancer and 300,000 deaths, and 80% of all the cervical cancer cases occur in developing regions [2]. Human papillomavirus (HPV) infection, particularly HPV 16 and HPV 18, and immunosuppression, smoking, pregnancy history and long-term use of oral contraceptives are the most critical risk factors for developing cervical cancer [3]. The techniques of cervical cancer screening such as the papanicolaou and HPV detection have been greatly improved in the past decades [4]. The infection of high-risk HPV does not necessarily lead to cervical cancer, suggesting that HPV infection is a principal but not a decisive cause of cervical cancer [5]. Study found that cervical cancer could develop independently by genetic changes with altered expressions of oncogenes or tumor suppressor genes or together with HPV infection [6]. The five-year survival rate for cervical cancer patients with early detection of cervical cancer is 92% [4], but the chance drops sharply for patients with tumor spreading to surrounding tissues or other distant organs. Therefore, the early detection of cervical cancer has a strong significance in cervical cancer intervention and treatment.

Copy number variation (CNV) is defined as a variation in genetic structure, usually between increase or decrease of 1kB-3 Mb at the copy number of a genome fragment [7]. Copy number amplification or deletion in cancer genome often leads to the inactivation of tumor suppressor genes or the expressions of oncogenes that have important effects on cell functions, including cell adhesion and recognition [8]. For example, the increase in the copy number of PD-L1 gene has been found to be related to the anti-PD-1/PD-L1 treatment of locally advanced cervical cancer [9]. HPV16 and HPV18 genome copies are associated with the grade of cervical lesions [10]. CNV not only refers to tumor biology, but also have effects on some complex immune diseases. Patients with copy number abnormalities of CCL3L1 are more susceptible to HIV/acquired immunodeficiency syndrome (AIDS) [11]. CNV of human Fcgr3 gene is determinant of glomerulonephritis [12]. At present, a large number of studies focused on messenger RNAs (mRNAs) and investigated the phenotypic changes and tumor progression caused by CNVs, while only a few studies were conducted to analyze the regulatory relationship between CNVs and non-coding RNAs, especially long non-coding RNAs (lncRNAs).

LncRNAs are defined as RNA transcripts with more than 200 base pairs in length and are a major class of ncRNA [13]. Abnormally expressed lncRNAs are closely related to complex human diseases, especially in tumors [14]. Dysfunctions of lncRNAs contribute to the development, progression and metastasis of cancers [15]. For example, lncRNA ARAP1-AS1 promotes the translation of the proto-oncogene c-Myc by isolating PSF/PTB dimers in cervical cancer, thereby promoting tumorigenesis and metastasis [16]. LncRNA NKILA inhibits proliferation and promotes the apoptosis of cervical squamous cells by down-regulating miRNA-21 expression [17]. LncRNA SBF2-AS1 enhances cervical cancer progression through regulating miR-361-5p/FOXM1 axis [18]. Expression profile shows highly abnormal expressions of lncRNAs in cancer, suggesting that lncRNAs could serve as a biomarker for predicting clinical outcomes [19].

High-throughput technologies allow histological studies to interrogate thousands of manufacturers with similar biochemical properties (e.g., RNA transcriptomes). Monolayer "histology" provides only limited insight into the biological mechanisms of diseases. In genome-wide association studies, although a great number of single-nucleotide polymorphisms have been identified for complex diseases and traits, the functional implications and mechanisms of the loci of interest remain largely unknown. In addition, genomic variation alone cannot fully explain changes in disease risk over a lifetime; DNA, RNA, proteins, and metabolites often play complementary roles and perform certain biological functions together. Such complementary effects and synergies between genomic layers in a life course can only be obtained through comprehensive studies of multiple molecular layers [20].

In this study, based on mRNA expressions, DNA methylation, and DNA copy number, we identified three molecular subtypes related to the prognosis of patients with cervical cancer. Differentially expressed mRNAs and lncRNAs in the three molecular subtypes were analyzed and examined to analyze the functions of co-expressed lncRNAs. Although CNVs play important role in transcriptional regulation, it was unclear whether CNV is systematically related to the expressions of lncRNAs in cervical cancer. By analyzing the copy number profile of lncRNAs across the genome, we carefully examined these abnormal lncRNAs induced by copy number amplification or deletion. In addition, Kaplan–Meier (KM) survival analysis was conducted to assess the prognostic performance of the lncRNAs with copy number abnormalities. Finally, a prognostic model was established to predict the survival of cervical cancer patients. This study aimed to identify CNV-related lncRNAs that can better predict cervical cancer prognosis.

## Methods

### Data download and processing

Methylation data, RNA-seq data, CNV, whole exome sequencing (WES) mutation data and sample follow-up information of Illumina Infinium 450 k Human DNA methylation Beadchip v1.2 platform for cervical cancer were downloaded from The Cancer Genome Atlas (TCGA) database (https://tcga-data.nci.nih.gov/) [21]. All the samples were collected before the first treatment. RNA-seq counts were converted into TPM (TranscriptsPerKilobase of exonmodel per Million mapped reads) expression profile data. Finally, expression profile data sets incorporating 304 cancer tissue samples and 3 para-cancer samples were obtained. According to the GeneCode v33 GTF [22], the expression profiles of 14,851 sense-intronic, sense-overlapping, antisense, processed-transcript, or primer-overlapping lncRNAs were acquired when gene type was defined as the lncRNA. When gene type was defined as protein-coding genes (PCGs), the expression profiles of 19,611 PCGs were obtained. For methylation data, CpG probes with the presence of NA expression in each sample were removed. At the same time, according to the cross-reactive site provided by Chen et al. [23], the CpG sites with cross-reactive in the genomes or unstable genome methylation sites were all removed, that is, the CpGs and single nucleotide sites on the sex chromosome were removed. In this way, a total of 372,137 CpGs sites were finally obtained. A total of 304 samples of copy number variation data were acquired after the removal of germline CNV data. Single nucleotide mutation data processed by MuTect software and clinical follow-up information of 307 cervical cancer samples were downloaded. A total of 292 primary tumor samples before the first treatment with complete data of RNA-seq, SNV, and methylation were detected for multi-omics clustering analysis. Meanwhile, standardized expression profile data GSE19711 containing 300 cervical cancer samples was download from the Gene Expression Omnibus (GEO) database (http://www.ncbi.nlm.nih.gov/geo/), with Illumina HumanHT-12 WG-DASL V4.0 R2 expression beadchip platform as an external validation. Sample information in TCGA data and GEO dataset are shown in Table 1.

**Table 1:** Clinical features of the data set

Characteristics	TCGA-Set				GSE44001(N = 300)
	Common Samples	RNA-seq(N = 304)	Methylation(N = 304)	CNV(N = 340)	
Survival event					
Dead	67	72	71	67	38
Live	225	232	233	235	262
Pathological_T					
T1	134	140	140	139	NA
T2	69	70	71	71	NA
T3	20	21	20	21	NA
T4	8	10	10	8	NA
Pathological_N					
N0	127	133	133	133	NA
N1	56	61	60	58	NA
N2	0	0	0	0	NA
N3	0	0	0	0	NA
Pathological_M					
M0	108	114	116	111	NA
M1	10	11	10	10	NA
Pathological_Stage					
Stage I	40	44	44	42	258
Stage II	55	56	57	57	42
Stage III	45	46	45	46	0
Stage IV	19	22	21	19	0
Pathological_Grade					
G1	18	19	18	18	NA
G2	128	134	135	133	NA
G3	113	118	118	118	NA
G4	1	1	1	1	NA
Age					
> = 60	56	59	59	56	NA
< 60	236	245	245	246	NA

### Prognostic molecular subtype identification based on multi-omics

The iClusterPlus [24], which is developed for comprehensive cluster analysis of multiple types of genomic data, is an enhanced version of iCluster. In this study, iClusterPlus was used to identify molecular subtypes based on DNA methylation, CNVs and transcriptome data. Specifically, we first analyzed the effects of PCGs, CNVs, and methylation on the prognosis of cervical cancer. Then the prognostic-related characteristics of DNA methylation, CNVs and transcriptome were examined by establishing Univariate COX proportional risk regression model. For potentially relevant features, the significance threshold was set as p < 0.05. Next, DNA methylation, CNVs and transcriptome samples were integrated to extract the data corresponding to the features related to the cancer prognosis. The R software package iClusterPlus [25] in R software package was further used for cluster analysis, The copy number data were segmented using the CBS algorithm^21^. The segment means were used as the input for integration to reduce the noise level, and the different histological data were further z-transformed and clustered using the K-means clustering algorithm, with the classification results of k = 2–10. Euclidean distance was used to assesses the distance of difference between samples. The classification results with the least difference within the group and the greatest difference among the groups were selected and employed to determine the molecular subtypes of cervical cancer prognosis.

### Subtype identification based on differentially expressed lncRNAs and PCGs

We used the R package DESeq2 [26] to identify lncRNAs and PCGs with subtype differences. Firstly, after removing the genes with an average count of < 5 in the expression profile, the differences of each subtype were compared. Other samples outside the subtype were taken as the control group, with the threshold of foldchange greater than twice and FDR < 0.05. Furthermore, the absolute value of the difference multiple was used as the rank order, and Gene Set Enrichment Analysis (GSEA) was applied to detect the distribution of DE-lncRNAs. The ncRNAs closely related to cervical cancer were downloaded from LncRNADisease [27], with Lnc2Cancer [28] database serving as the background for the comparison of the relationship between de-lncRNAs and cervical cancer.

### Identification of enriched lncRNA modules by weighted gene co-expression network analysis

Weighted gene co-expression network analysis (WGCNA) package [29] in R was used to construct a scale-free co-expression network for the differentially methylation sites (DMPs). Pearson's correlation matrices and average-linkage method were both conducted for differential expression (DE)-PCGs/lncRNAs. β was a soft-thresholding parameter that emphasizes strong association between PCGs and penalized weak association. Then, the adjacency was converted into a topological overlap matrix (TOM), which was defined by the sum of its adjacency with all other DE-PCGs/lncRNAs for network DE- PCGs/lncRNAs ration, and the corresponding dissimilarity (1-TOM) was calculated. *p* < 0.05 was set as the threshold to identify the modules with significant enrichment in DE-lncRNAs. Finally, based on the genes in the DE-lncRNAs enrichment module, R package clusterprofiler was used to perform Kyoto Encyclopedia of Genes and Genomes (KEGG) pathway enrichment analysis.

### Genomic copy number anomalies and their relationship to lncRNAs were analyzed by GISTIC algorithm

Genes targeted by somatic cell copy number change (SCNA) play an important role in tumorigenesis and cancer therapy. Here, we used GISTIC2.0 [30] software to define CNVs extracted from of all the genes in the 292 cervical cancer samples were related to lncRNAs. Copy number > 1 or < -1 was defined as copy number amplification and deletion, respectively. According to FDR < 0.05, the boundaries of the regions with peak amplification or deletion limits identified by GISTIC 2.0 were determined with at least 95% confidence to include the target gene/lncRNA(s). Samples with lncRNA expression profiles were selected, lncRNA expression profiles and copy numbers were calculated by Spearman's rank correlation coefficient, and random differences in the distribution of correlation coefficients were compared. Finally, the lncRNAs with CNV in more than 25% of the samples were identified, and their expression differences were compared in copy amplification and copy deletion samples.

### Identification of lncRNA prognostic markers with abnormal copy number and establishment of gene signature based on lncRNAs

We systematically analyzed the CNV of these de-lncRNAs on the basis of molecular subtypes, and selected lncRNAs with a proportion of abnormal copy number higher than 15%. Univariate cox was used to analyze the relationship between these lncRNAs and overall survival (OS). The threshold was defined as p < 0.01 to screen lncRNAs significantly related to the prognosis of cervical cancer. In addition, least absolute shrinkage and selection operator (LASSO) Cox was used for analyzing the expression profiles of these lncRNAs for feature selection [31]. The tenfold cross-validation was used to construct the model. Finally, the Multivariate Cox survival analysis was performed, and the lncRNAs with minimal Akaike information criterion (AIC) value was considered as the final prognostic markers to establish the risk score model:

$$RiskScore={\sum }_{k=1}^{n}{Exp}_{k}*{{e}^{HR}}_{k}$$

where N is the number of prognostic lncRNAs, ${Exp}_{k}$ is the expression value of prognostic lncRNAs, and ${{e}^{HR}}_{k}$ is the estimated regression coefficient of lncRNAs in the multivariate Cox regression analysis.

### Functional enrichment analysis

GSVA [32] was performed using the R package on C2 Canonical pathway gene set collection that contained 1320 gene sets obtained from MsigDB database. The enrichment scores of each sample in these gene sets were analyzed using single sample GSEA, and the association between the enrichment scores of each gene sets and RiskScore was further calculated by Spearman's rank correlation coefficient. KEGG pathway with the absolute correlation coefficient greater than 0.4 and FDR < 0.01 was selected.

### Performance comparison of the lncRNA signatures

To determine the independence of the lncRNA signature, we performed single-factor and multi-factor Cox regression to analyze the relationships among T, N, M stage, age and risk score and prognosis. Furthermore, we compared the newly established lncRNA signatures with four recently reported prognostic risk models, which were 4-lncRNAs signature by sun et al. [33], 10-lncRNA signature by Shen et al. [34], 9-lncRNAs signature by Mao et al. [35], and 6-lncRNA signature by Luo et al. [36]. To ensure the model comparability, we calculated the risk score of each cervical cancer sample in the TCGA dataset using the same method based on the corresponding genes in these 4 models. Moreover, the receiver operating characteristic (ROC) of each model was determined and the risk score was calculated based on the median risk score. The samples were divided into Risk-H and Risk-L groups, and the prognostic differences between the two groups of samples were calculated. Furthermore, we compared these four models with restricted mean survival, concordance index (C-index), and decision curve analysis (DCA) of the lncRNA signature.

### Other statistical descriptions

Except for special instructions, the normality of variables was examined by Shapiro–Wilk normality test. Significance of the normal distribution variables was estimated by the unpaired Student's t test between two groups, and the non-normal distribution variables were analyzed by the Mann–Whitney U test. Kruskal–Wallis test and one-way analysis of variance served as non-parametric and parametric methods, respectively, to analyze differences among multiple groups. Correlation coefficient was calculated by Spearman's rank correlation coefficient. Two-sided Fisher exact test was performed to determine contingency tables. The p value was converted into FDR by Benjamini–Hochberg method. Survival curves of each subgroup in the dataset were plotted by Kaplan–Meier (KM) method. Logrank test was used to determine the statistical significance of the differences, which was defined as *p* < 0.05. All the analyses were performed in R 3.4.3 using default parameters unless otherwise specified.

## Results

### Identification of prognostic molecular subtypes through comprehensive analysis of DNA methylation, CNVs and transcriptome

**Fig. 1:**
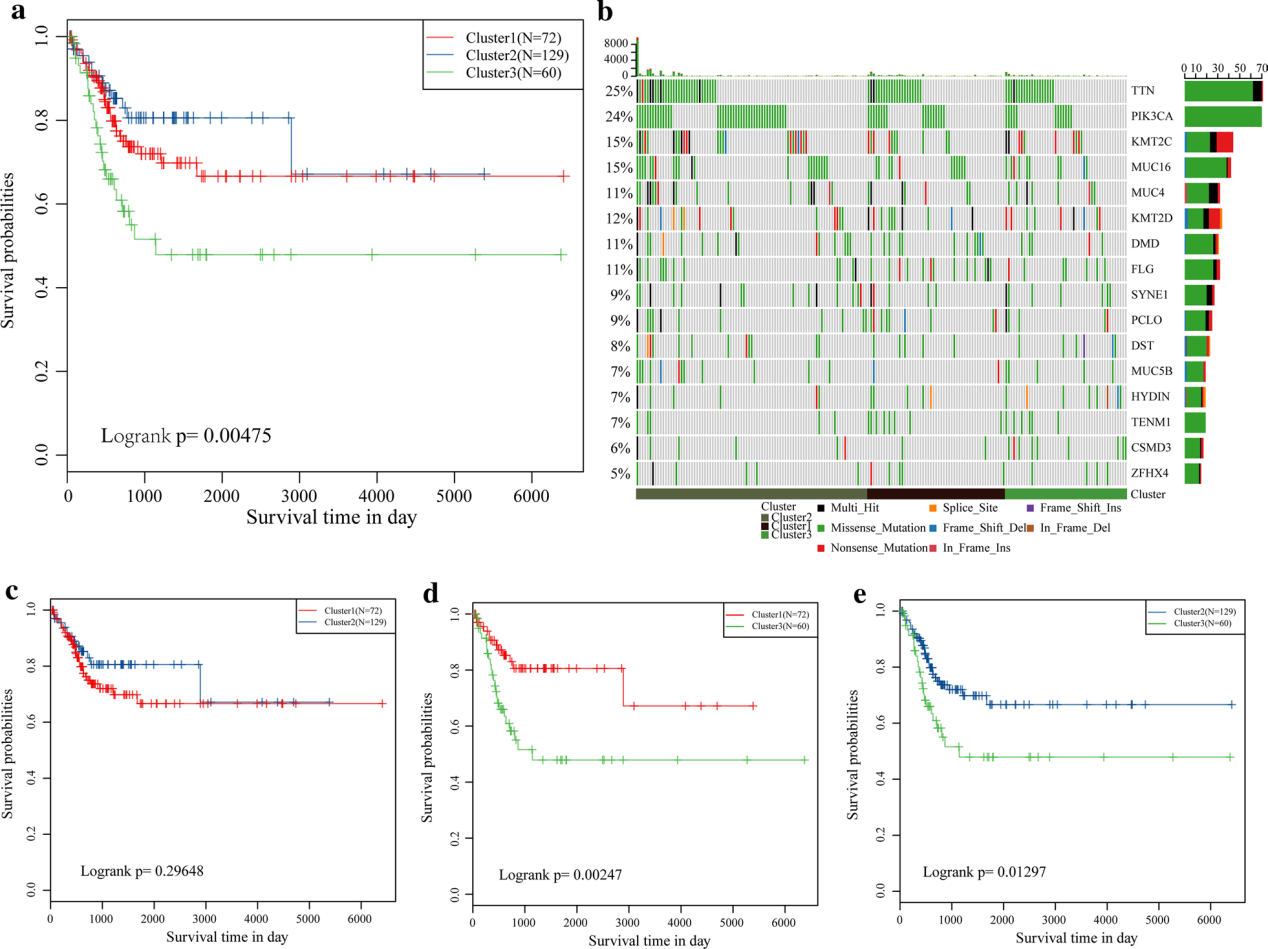
Prognostic differences and molecular characteristics of the three molecular subtypes. **a** The total survival and prognosis of the three subtypes were different by KM curves. **b** The mutation distribution of high-frequency mutation genes in the three molecular subtypes of cervical cancer. The horizontal is molecular subtypes, ordinate (left) is the mutation frequency of the gene in each sample, ordinate (right) is mutation genes and colors in heat-map are different mutation types. **c** KM curve results showed there is no difference in prognosis between Cluster1 and Cluster2. d KM curve results showed there is significantly difference in prognosis between Cluster1 and Cluster3. **e** KM curve results showed there is significant difference in prognosis between Cluster2 and Cluster3

To identify the prognostic molecular subtypes of cervical cancer, genes showing PCGs, CNV and methylation with prognostic significance were screened based on univariate Cox proportional risk model, with a threshold of p < 0.05. Finally, 3897 genes, 4897 CNV regions and 20,144 CpG sites with significant prognostic association were obtained. Next, iClusterPlus was used for multi-omics cluster analysis, and identified three molecular subtypes, which were Cluster1 (N = 77), Cluster2 (N = 142) and Cluster3 (N = 73). The results of multi-omics clustering were compared with those obtained by separate hierarchical clustering (Fig. S1A), and the results showed that the three molecular subtypes determined by multi-omics clustering had some consistency with those obtained by single-omics clustering. For example, the methylation clustering results of Cluster1 and Cluster2 subgroups overlapped with those of multi-omics patients with Cluster1, suggesting that the molecular subtypes identified by multiple histologies combining the molecular characteristics of multiple histological data are richer in terms of information dimensions than those of individual histology, and that no one histology could reproduce the molecular subtypes identified by multiple histologies alone. The three subtypes had significant prognostic differences (p = 0.0047) (Fig. 1a), among them, Cluster1 showed the most favorable prognosis, while the Cluster3 had the worst prognosis. There was no significant difference in prognosis between the Cluster1 and Cluster2 groups (Fig. 1c), but the prognosis of Cluster3 was greatly worse than that of Cluster1 (p = 0.00247) and Cluster2 (p = 0.01297) (Fig. 1d, e). A total of 16 were obtained by the detection of the top10 high-frequency mutated genes of each subtype (Fig. 1b). The distribution of these genes was similar in all three subtypes, suggesting a high degree of consistency in the genes associated with the most common mutations in the three subtypes. In addition, TTN, PIK3CA, KMT2C, and MUC4 with higher mutation rates may suggest that these genes play a key role in canceration. The findings here indicated that subtype classification based on PCGs, methylation, and CNVs could predict the prognosis of patients with cervical cancer and have certain regulatory relationships at the levels of genome, epigenome, and transcription.

### Distribution of differentially expressed lncRNAs and PCGs in the three subtypes

**Fig. 2:**
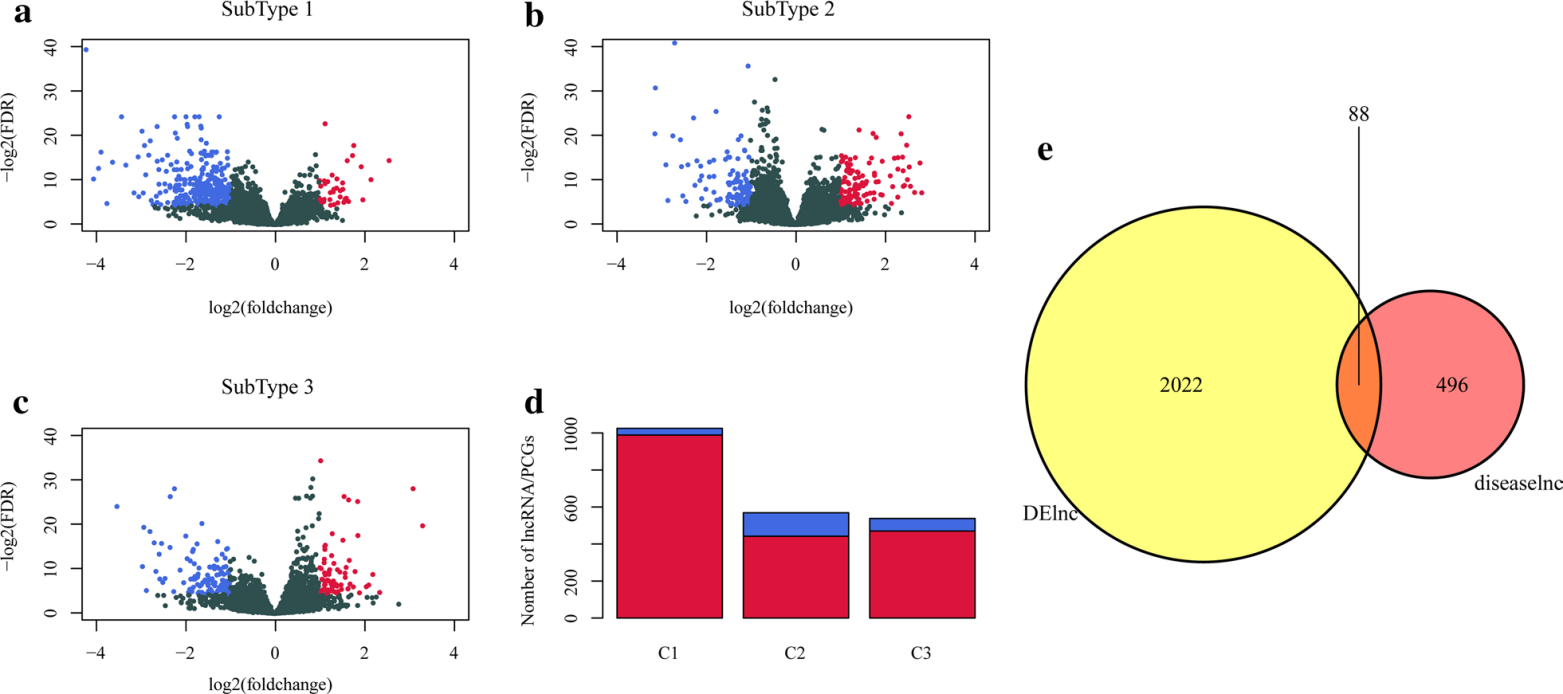
Volcano gram and distribution of differentially expressed lncRNAs. **a** Volcano plot of differentially expressed lncRNAs in Cluster1. **b** Volcano plot of differentially expressed lncRNAs in Cluster2. **c** Volcano plot of differentially expressed lncRNAs in Cluster3. Red is up-regulated lncRNAs, blue is down-regulated lncRNAs. **d** The number of differentially expressed lncRNAs and PCGs genes in the three subtypes. Red is differentially expressed lncRNAs and blue is differentially expressed PCGs. **e** Venn diagram of the intersection of subtype-different lncRNAs and disease-related lncRNAs

The differentially expressed lncRNAs and PCGs in three subtypes were determined. We found the small number of differentially expressed lncRNAs in both Cluster2 samples and Cluster3 samples. A total of 617 lncRNAs and 1395 PCGs were obtained from the three subtypes (Additional file 1: Table S1). The volcano map data of the differential expressions of the lncRNAs showed that in the three subtypes the number of up-regulated lncRNAs was generally smaller than those down-regulated (Fig. 2a-c). Moreover, the numbers of differentially expressed PCGs was much larger than that of lncRNAs (Fig. 2d). Finally, 584 lncRNAs closely related to cervical cancer were downloaded from LncRNADisease and Lnc2Cancer databases. We found that there was an obvious intersection between differentially expressed lncRNAs in the three subtypes and cervical cancer-related lncRNAs (p < 0.0001) (Fig. 2g). Those data suggested that lncRNAs may play an important role in the heterogeneity and progression of cervical cancer.

### Identification of differentially co-expressed lncRNAs-PCGs and functional modules for lncRNAs enrichment in the three subtypes

**Fig. 3:**
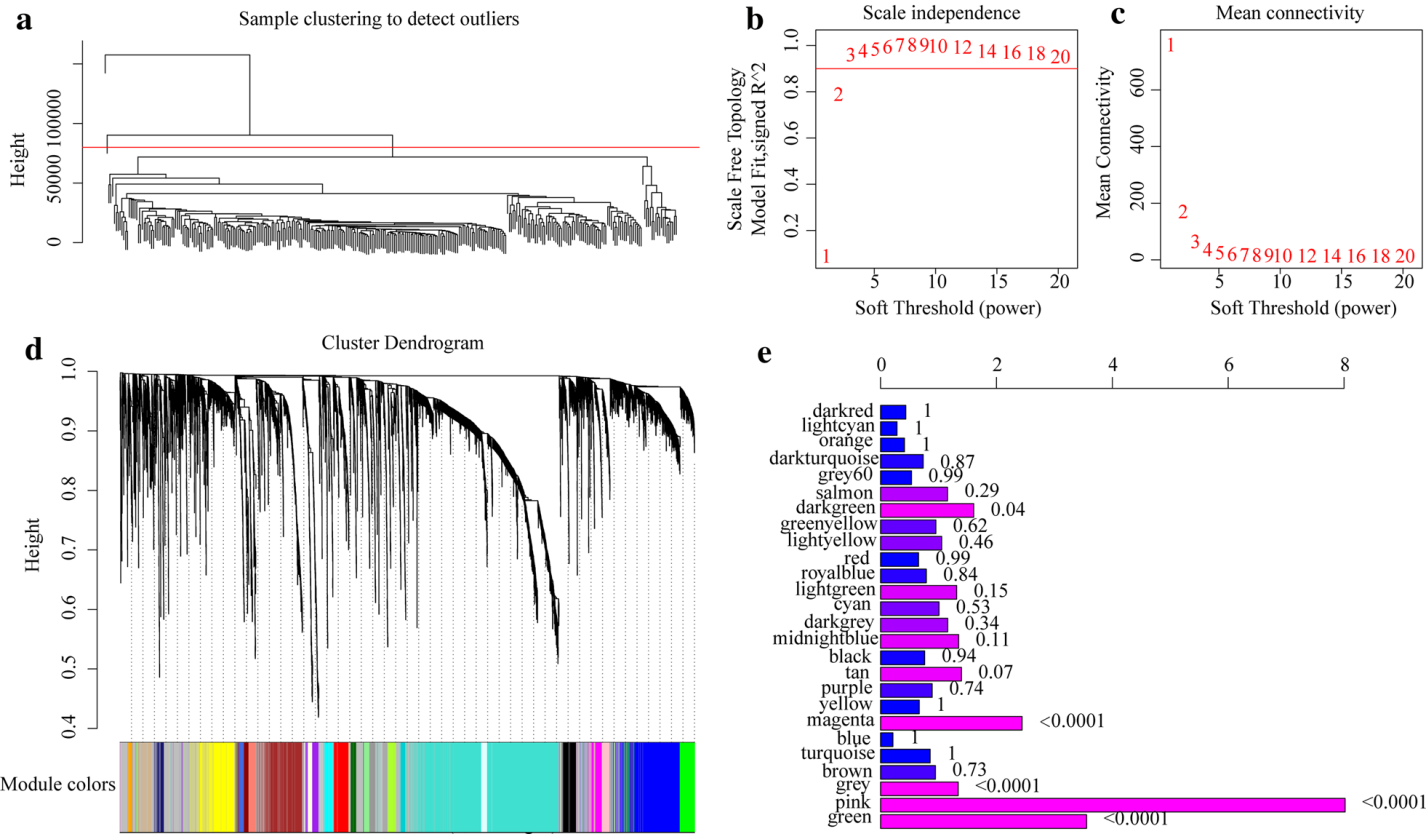
The co-expression modules of lncRNAs and PCGs were identified by WGCNA analysis. **a** The hierarchical clustering analysis of samples was based on the expression profiles of lncRNAs and PCGs. **b** Analysis of the scale-free fit index for various soft-thresholding powers (β). **c** Analysis of the mean connectivity for various soft-thresholding powers. **d** Dendrogram of all differentially expressed genes clustered based on a dissimilarity measure (1-TOM). **e** Relative multiples of lncRNAs ratio and PCG ratio in 25 modules. The value on the right is the significant p value, the horizontal axis is the multiple of the ratio of lncRNAs to PCG in the module, and the vertical axis is the module

**Fig. 4:**
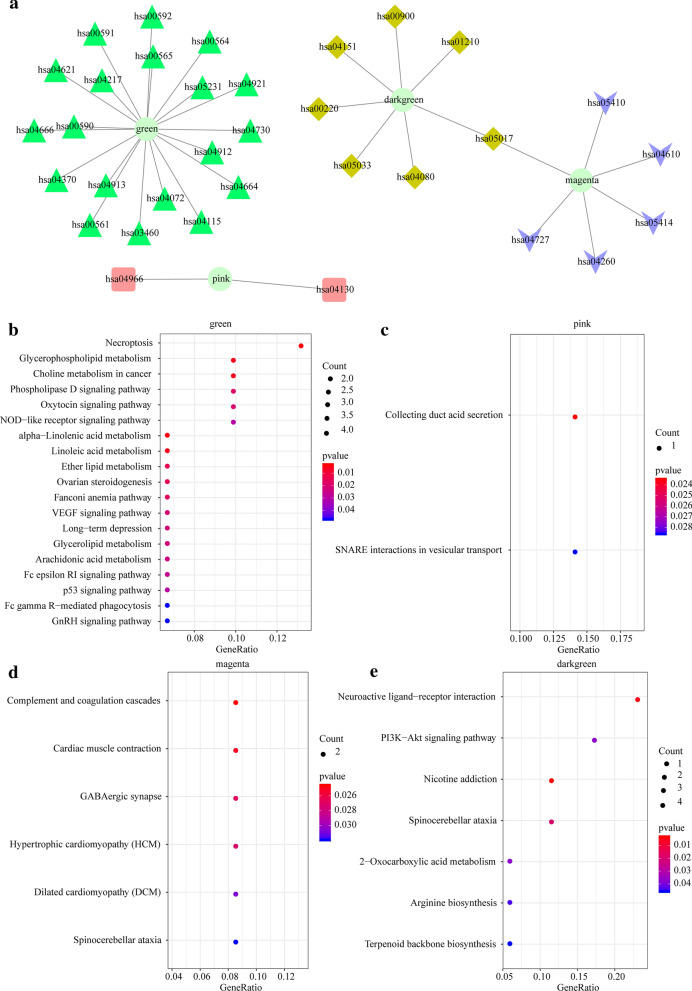
KEGG enrichment analysis of four lncRNA enrichment modules. **a** Network relationship of enrichment results of the four modules (green, pink, magenta and darkgreen). **b** Enrichment results of gene KEGG in green module. **c** KEGG enrichment results of genes in pink module. **d** KEGG enrichment results of genes in magenta module. **e** KEGG enrichment results of genes in darkgreen module. The color from red to blue represents the p value from large to small, the size of the circle represents the number of genes in the enrichment pathway, with a larger circle representing more gene data

**Fig. 5:**
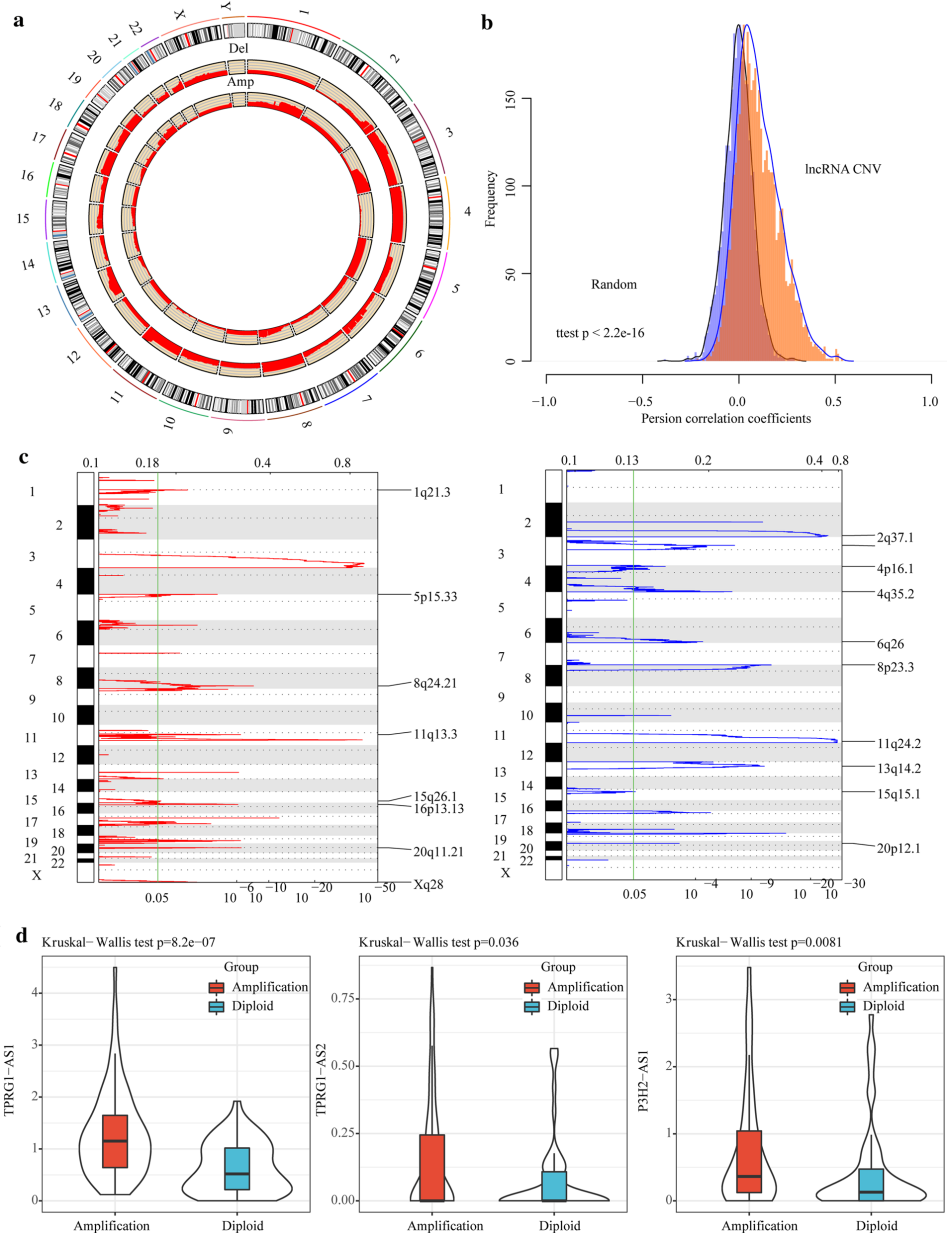
Abnormal expressions of lncRNAs were positively correlated with abnormal copy numbers. **a** Distribution of lncRNA copy number amplification and deletion in genome. **b** The correlation distribution between lncRNA expressions and CNVs, light blue represents the distribution under random conditions, orange represents the distribution under actual conditions, t-test was used to examine the difference. **c** The lncRNAs located in the focal CNA peaks are cervical cancer-related. False-discovery rates and scores from GISTIC 2.0 for alterations (x-axis) are plotted against genome positions (y-axis); dotted lines indicate the centromeres. The deletions (right, blue) and amplifications (left, red) of lncRNAs genes are also shown. The green line represents 0.05 (FDR) as cut-off point that determines significance. **d** The expressions of lncRNAs in the samples with copy amplification are significantly higher than that in the samples with normal copies

To identify differentially co-expressed lncRNA-PCGs, based on the differential expression profiles of lncRNAs and PCGs, outlier samples were removed by hierarchical clustering analysis. The linkage method was chosen as the complete method, and the clustering distance was calculated by Euclidean clustering, and those clustering distances exceeding five times the standard deviation (80,572.36) were considered as outliers and were rejected. We finally obtained a total of 307 samples (Fig. 3a). WGCNA was conducted for building expression network, with β = 3 (scale-free R 2 = 0.92) of power as a Soft Thresholding to ensure a scale-free network (Fig. 3b, c). Here, a total of 26 modules were screened (Fig. 3d). The numbers of lncRNAs and PCGs in each module are shown in Additional file 2: Table S2. By analyzing the enrichment level of lncRNAs in each module, the number of lncRNAs and PCGs in each module was first counted, then the significance of lncRNA enrichment in each module was calculated using Fisher exact test with all DE-lncRNAs and DE-PCGs as background. The following four modules with significant enrichment of lncRNAs were identified: green, pink, magenta, and darkgreen (Fig. 3e). The KEGG pathway enrichment analysis of the PCGs in the four modules showed that the PCGs in these four modules were enriched to a total of 41 KEGG pathways (Fig. 4a). The pathways enriched by the four modules each showed limited intersection, and they tended to enrich to different pathways, suggesting that different modules may have different functions. The green module was found enriched to 19 KEGG pathways, and was mainly enriched to p53 signaling pathway, cAMP signaling pathway, Glucagon signaling pathway and some other pathways significantly related to tumorigenesis, development, tumor metabolism of cervical cancer (Fig. 4b). The pink module was enriched to the collecting duct acid secretion and SNARE interactions in vesicular transport pathway (Fig. 4c). As shown in Fig. 5D, the magenta module was enriched to 6 KEGG pathways, which are mainly the pathways related to cardiomyopathy such as Hypertrophic cardiomyopathy (HCM) and Dilated cardiomyopathy (DCM) (Fig. 4d). It is known that advanced cancers can easily induce great changes in metabolism, promote cardiac atrophy, and heart failure [37]. As shown in Fig. 5e, the darkgreen module was enriched to 7 KEGG pathways, mainly to PI3K-Akt signaling pathway, Nicotine addiction and other pathways related to tumorigenesis and development (Fig. 4e). These results indicated that lncRNAs may be directly or indirectly involved in important pathways of the tumorigenesis and development of cervical cancer.

### Abnormal expressions of lncRNAs were relevant to CNVs

To analyze the relationship between lncRNA expressions and CNVs, lncRNAs copy number data were extracted from 292 cases of cervical cancer cases obtained from the TCGA, with copy number greater than 1 as the threshold of copy number amplification and less than -1 as the copy number deletion threshold. The ratio of copy number amplification and copy number deletion of each lncRNAs and its distribution in the genome were analyzed (Fig. 5a). Hwere, copy number deletion and copy number amplification showed different distributions on different chromosomes. For example, most copies of chromosome 3, 4, 8, 11 and 17 were absent, while some copies of chromosome 1 and 3 were increased. The association distribution between the expression profile of lncRNAs and copy number demonstrated an overall positive related trend, and the distribution in the actual situation was significantly larger than that in the random case (p < 2.2e-16) (Fig. 5b). The frequently changing regions in the genome of cervical cancer patients were detected using GISTIC algorithm, and the data revealed many regions with significant copy number amplification or deletion of lncRNAs (Fig. 5c), suggesting that the abnormal copy number of lncRNAs may be related to the occurrence and development of cervical cancer. A total of 3 lncRNAs with a copy number ratio of more than 25% in each sample were identified, and their expression differences in copy number amplification/deletion and normal copied samples were analyzed. The data showed that the expressions of lncRNAs in the samples with copy amplification were significantly higher than that in the samples with normal copies (Fig. 5d), indicating that the abnormal expressions of lncRNAs were related to the abnormal copy number.

### Identification of lncRNA prognostic markers with abnormal copy number in cervical cancer patients and establishment of a lncRNA signature

**Fig. 6:**
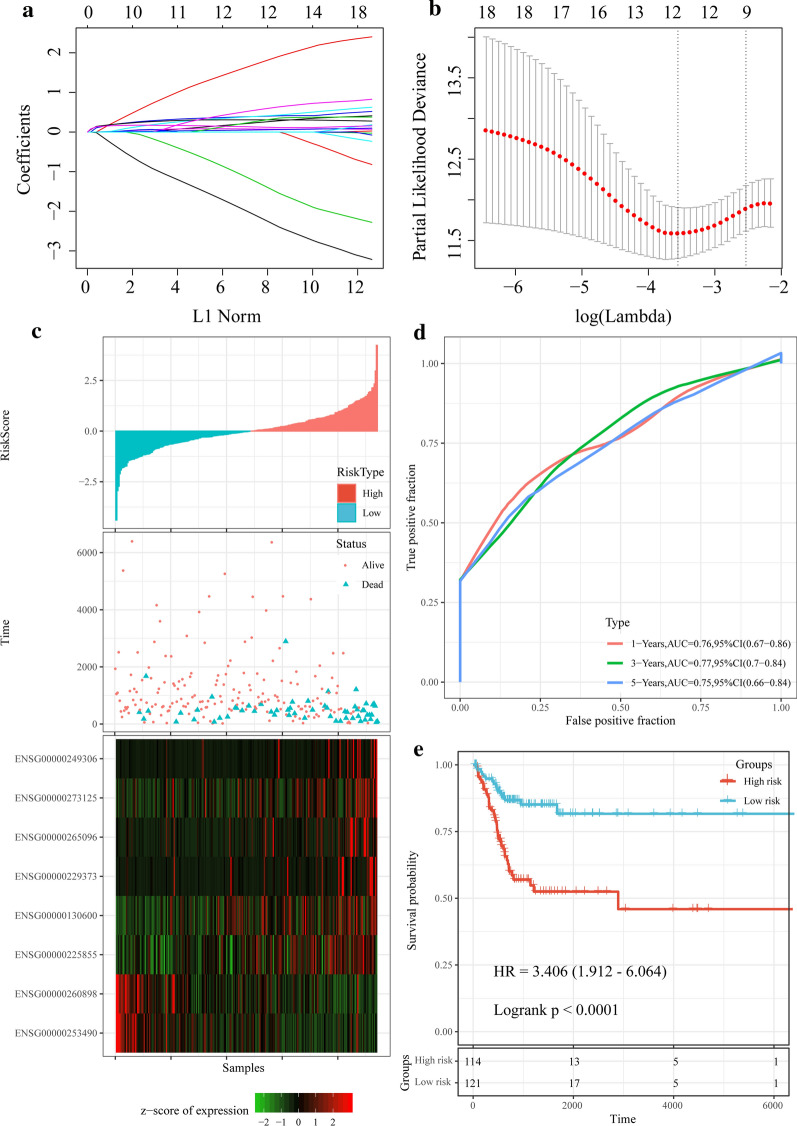
Screening of lncRNA prognostic markers and establishment of prognostic models. **a** The number of genes is increasing as lambda increases, the horizontal axis represents the log value of the independent variable lambda, and the vertical axis represents the coefficient of the independent variable. **b** Confidence interval under each lambda. **c** Risk score, survival time, survival status and expression of the 8-lncRNA signature in the training set. **d** ROC curve and AUC of the 8-lncRNA signature in training set. **e** KM survival curve distribution of the 8-lncRNA signature in the training set

A total of 575 lncRNAs with CNV greater than 15% were selected. Univariate Cox analysis was performed to examine the relationship between these lncRNAs and OS, and we found that 41 lncRNAs significantly related to the cancer prognosis *p* < 0.01 (Additional file 3: Table S3). LASSO Cox regression analysis was further performed to analyze the expression profiles of these lncRNAs. The change trajectory of each independent variable (Fig. 6a) demonstrated that the number of independent variable coefficients close to 0 gradually increased with the gradual increase of lambda (Fig. 6b). The model was built using tenfold cross-validation, and the confidence interval under each lambda was analyzed. When lambda = 0.0285, we found that the model reached the highest performance, and there were 12 lncRNAs, which could serve as the potential prognostic markers. Furthermore, multivariate Cox survival analysis was performed, and 8 lncRNAs (Additional file 4: Table S4) with the lowest AIC value (AIC = 546.05) were obtained as the final prognostic markers to establish a risk regression model:

RiskScore = 0.486*exp^ENSG00000225855^ + 2.707*exp^ENSG00000273125^ + 0.573*exp^ENSG00000249306^−2.601*exp^ENSG00000253490^ + 0.096*exp^ENSG00000130600^ + 0.768*exp^ENSG00000229373^−3.111*exp^ENSG00000260898^ + 0.684*exp^ENSG00000265096^.

The relationship between risk score and lncRNA expressions was shown in Fig. 7c. As the risk score increased, the mortality rate of the samples also increased. 6 high-expressed lncRNAs associated with high risk score were a risk factors, while 2 low-expressed lncRNAs associated with high risk score were protective factors (Fig. 6c). One-year, three-year and five-year of ROC analysis and prediction showed that the model had a high AUC area, and they all had an AUC above 0.75 (Fig. 6d). Finally, Zscore of the risk score was calculated to divide the samples with a risk score higher than zero into high-risk group (N = 114) and with a score lower than zero into low-risk groups (N = 121). Interestingly, the samples in the high-risk group showed significantly worse prognosis than those in the low-risk group (p < 0.0001, HR = 3.406, 95% CI: 1.912–6.064) (Fig. 6e).

In addition, 50% of the 292 samples were randomly selected and repeated one thousand times. The model was applied to the prognostic prediction of these one thousand samples, and the prognostic significance p-values were calculated for each calculation (Additional file 5: Fig. S1B). The prognostic predictive power in these samples was observed to show significant differences in the prognostic significance p-values for each of the one thousand random samples. In addition, we also analyzed the correlation between 8 lncRNAs (Additional file 5: Fig. S1C), and only a few were significantly correlated, as expected there was no covariance among them.

### Prognostic model validation and functional analysis of the 8-lncRNA model

To verify the performance of the 8 CNV-related lncRNAs in predicting the prognosis of cervical cancer, the risk scores of each sample in all TCGA data sets were calculated according to the expressions of the samples, and the predictive classification efficiency of 1-year, 3-year, and 5-year AUC was determined. The results showed that the model had a high AUC line area above 0.75 (Additional file 6: Fig. S2a). The samples were divided into high- and low-risk groups according to the threshold, and we found that the prognosis of the high-risk group was significantly worse than that of the low-risk group (p < 0.0001, HR = 3.133, 95%CI:1.854—5.291) (Additional file 6: Fig. S2a). To further verify the robustness of the model, the GSE44001 data of GPL14951 platform was downloaded. The average AUC of 1-year, 3-year, and 5-year ROC was all higher than 0.6 (Additional file 6: Fig. S2c). Moreover, we also assessed the prognosis of the samples in high-risk group and low-risk group, and observed that the prognosis of the high-risk group was significantly worse than that of the low-risk group (p = 0.036, HR = 1.957, 95%CI: 1.032–3.707) (Additional file 6: Fig. S2d). These data indicated that the performance model of 8-lncRNA signature model had a great robustness. To investigate the relationship between Riskscore and biological functions of different samples, risk score was correlated with the enrichment score of KEGG pathways in each sample. KEGG pathways with a correlation greater than 0.4 and FDR < 0.01 contained 20 pathways (Additional file 6: Fig. S2e). Interestingly, these 20 pathways were negatively correlated with Riskscore and mainly included T CELL RECEPTOR SIGNALING PATHWAY, B_CELL_RECEPTOR_SIGNALING_PATHWAY, CYTOSOLIC_DNA_SENSING_PATHWAY, CYTOKINE_CYTOKINE_RECEPTOR_INTERACTION and some other immune-related pathways. The results suggested that the samples from the high-risk group and the low-risk group may have different immune microenvironments, and that these lncRNAs may be involved in tumor progression by affecting immune-related pathways.

### Comparison of 8-lncRNA prognosis model with clinical features and the existing models

To identify the independence of the 8-lncRNA signature, the relationship between T, N, M stage, age, and risk score and prognosis was analyzed by Univariate and multivariate Cox regression analysis. Univariate Cox regression analysis showed that N stage, TNM stage and risk score were significantly related to survival (Additional file 7: Fig. S3a), However, multivariate Cox regression analysis found that only risk score (p < 0.0001, HR = 3.425, 95% CI: 1.922–6.104) was significantly correlated with prognosis (Fig. S3B). Thus, the data revealed that the 8-lncRNAs signature can serve as a prognostic predictor independent of clinical characteristics. Furthermore, we compared the 8-lncRNA signature with four recently reported prognostic-related risk models, namely, the 4-lncRNA signature by sun et al. [33], the 10-lncRNA signature by Shen et al. [34], the 9-lncRNA signature by Yu et al. [35], and the 6-lncRNA signature by Luo et al. [36]. In order to ensure the comparability of the models, according to the corresponding genes in these 4 models, the same method was used to calculate the risk score of each cervical cancer sample in the TCGA dataset and ROC of each model, and KM survival curve was plotted. Although the prognosis of the Risk-H and Risk-L group samples of the four models were significantly different, the AUC prediction accuracy of the four models was lower than that of our 8-lncRNA model (Additional file 7: Fig. S3C-F). The restricted mean survival of these four models was also compared with our 8-lncRNA model. We observed that the 8-lncRNA model was more accurate in predicting a longer follow-up time and the C-index was higher than the other four models (Additional file 7: Fig. S3g). Similarly, the DCA results showed that the risk score of the 8-lncRNA model developed in this study was far more indicative than the other four subtypes (Additional file 7: Fig. S3H). These results suggested that the 8-lncRNA signature is a new reliable prognostic marker independent of clinical stages.

## Discussion

With the rapid development of next-generation sequencing and mass spectrometry technology, the biological complexity of tumors and the genetic etiology of cervical cancer has been increasingly elucidated and developed. In this study, we identified three prognostic molecular subtypes of cervical cancer based on multidimensional omics analysis, and screened subtype-specific lncRNAs and PCGs. Based on weighted co-expression analysis of the type-specific lncRNAs, three subtypes were found significantly related to the metabolism, immunity and other pathways of cervical cancer, suggesting that the subtype-specific lncRNAs may play different roles in the occurrence and development of cervical cancer and have different effects on the tumor progression. The relationship between the expressions of these lncRNAs and copy numbers were systematically analyzed, and the results demonstrated that the expressions of these lncRNAs were highly correlated with copy number amplification and deletion. LncRNAs with copy amplification tended be high-expressed, while those with deletion tended to be low-expressed. Based on CNVs and lncRNA expressions, 8 lncRNAs were identified as the potential prognostic markers for cervical cancer. The 8-lncRNA signature showed an accurate predictive performance in both the training set and the verification set, and can therefore be used as an independent prognostic factor for cervical cancer. Compared with other existing lncRNA signatures, our 8-lncRNA signature showed more stable predictive performance and higher AUC.

Past studies have shown that integrating multiomics clustering, such as iCluster, intNMF, Similar Network Fusion (SNF), could reveal tumor heterogeneity and actual prognostic features. In this study, multidimensional data processing was performed using iCluster based on a joint latent variable model of cervical cancer. The most noticeable feature of iCluster is the combination of estimated unobserved variables such as copy number data, mRNA expression data, and methylation, also iCluster can reduce the dimensionality of the data set without changing the sample size. Our algorithm matrix had a cohort of 292 cervical cancer patients, and the genome and epigenome contained three omics data, including mRNA, CNV, and methylation, which showed unique molecular characteristics and prognostic relevance. The prognosis of the Cluster3 subtype was poor, while the results of the Cluster2 subtype were the most favorable. Recent studies found that CNVcor and methylation-related genes (METcor) are significantly co-regulated, moreover, the integration of CNVcor and METcor genes have identified three molecular subtypes in liver cancer [38]. This suggested the significance of establishing a comprehensive prognostic molecular subtype based on genome and epigenome. In this study, we compared mutation profiles of the three molecular subtypes, and discovered that TTN, PIK3CA, KMT2C and MUC4 mutations were more common than other genes. Noticeably, the PIK3CA mutation is related to the resistance of cervical cancer to the treatment [39]. MUC4, which is a transmembrane glycoprotein expressed higher in cervical dysplasia than in benign cervical epithelium, is also related to lymph node metastasis of cervical cancer [40].

The occurrence and development of cancerous changes are often associated with enormous genomic mutations, including small size mutations (SNPs) and CNVs, loss of copy number, duplication, and amplification. CNV is a hallmark of cancer and often causes abnormal copy numbers, including amplification, increase, loss, and deletion. CNV plays an important role in regulating the expressions of PCGs and lncRNAs and the activation of multiple signaling pathways. It is known that CNV has critical functions in the development of various tumors, such as ovarian cancer [41], breast cancer [42], endometrial cancer [42].

Although we identified potential lncRNAs predictive of the prognosis of cervical cancer from large samples by applying bioinformatics techniques, some limitations still exist in this study. Firstly, the sample lacked clinical follow-up information, thus, factors such as the presence of other health conditions were not considered during the identification of the biomarkers. Secondly, the results obtained by bioinformatics analysis alone were not convincing enough, which requires further experimental verification. Therefore, genetic and experimental studies with larger sample sizes and experimental validation are needed.

## Conclusion

To conclude, we identified prognostic-associated molecular subtypes by conducting Multi-omics analysis. 8 lncRNAs with abnormal copy numbers were determined as prognostic markers, and an 8-lncRNA prognostic layering system was developed. The 8-lncRNA signature showed a high AUC in both training and validation sets, and was independent of clinical features. Compared with clinical features, the 8-lncRNA classifier could greatly improve the accuracy of predicting survival risk. Therefore, this classifier can be used as a reliable molecular diagnostic model in evaluating the prognostic risk of patients with cervical cancer.

## Supplementary Information

**Additional file 1: Table S1.** Distribution of differentially expressed lncRNA and PCGs in three subtypes.

** Additional file 2: Table S2.** CGPs and lncRNA in 26 modules.

**Additional file 3: Table S3.** Prognostic information of 41 lncrnas with significant prognosis.

** Additional file 4: Table S4.** Information of 8-lncRNA signature.

** Additional file 5: Figure S1.** Advantage of multi-omics.** a** The results of multi-omics clustering were compared with those obtained by separate hierarchical clustering.** b**: Model was applied to the prognostic prediction of these one thousand samples, and the prognostic significance p-values were calculated for each calculation. **c** The correlation between 8 lncRNAs were analyzed.

**Additional file 6: Figure S2. ** Prognostic model validation and functional analysis of the 8-lncRNA model.** a** ROC curve of the 8-lncRNA model in all TCGA datasets. Abscissa means false positive fraction, ordinate means true positive fraction.** b** KM survival curve distribution of the 8-lncRNA model in the high- and low-risk groups in all TCGA datasets. Abscissa means time, ordinate means survival probability.** c** ROC curve of the 8-lncRNA model in GSE44001 dataset. Abscissa means false positive fraction, ordinate means true positive fraction.** d** KM survival curve distribution of the 8-lncRNAs in the high- and low- risk group in GSE44001 datasets. Abscissa means time, ordinate means survival probability.** e** KEGG Pathway was the most correlated with the 8-lncRNA model, and the circle size in the figure indicates the correlation.

**Additional file 7: Figure S3.** Comparison of the 8-lncRNA prognosis model with clinical features and the existing models** a** Forest characteristics of clinical features and risk score using univariate survival analysis.** b** Forest characteristics of clinical characteristics and risk score using multivariate survival analysis, and among them, orange-red represents a significant prognostic correlation.** c** ROC curve and KM curve of a 4-lncRNA signature in TCGA dataset.** d** ROC curve and KM curve of a 10-lncRNA signature in TCGA dataset.** e** ROC curve and KM curve of a 9-lncRNA signature in TCGA dataset.** f** OC curve and KM curve of a 6-lncRNA signature in TCGA dataset. Left: Abscissa means false positive fraction, ordinate means true positive fraction. Right: Abscissa means time, ordinate means survival probality. G: Comparison of restricted mean survival of five prognostic risk models. Abscissa means restricted mean survival, ordinate means percentiles of marker.** h** Comparison of decision curve analysis of the five prognostic risk models. Abscissa means threshold probability, ordinate means net benefit.

## Data Availability

The data that support the findings of this study are available from the corresponding author upon reasonable request.

## Abbreviations

CNV: Copy number variation
lncRNAs: Long non-coding RNAs
mRNAs: Messenger RNAs
PCGs: Protein-coding genes
LASSO: Least absolute shrinkage and selection operator ()
WES: Whole exome sequencing
TCGA: The Cancer Genome Atlas
GEO: Gene Expression Omnibus
GSEA: Gene Set Enrichment Analysis
WGCNA: Weighted gene co-expression network analysis
DE: Differential expression
TOM: Topological overlap matrix
KEGG: Kyoto Encyclopedia of Genes and Genomes
SCNA: Somatic cell copy number change
OS: Overall survival
AIC: Akaike information criterion
ROC: Receiver operating characteristic
C-index: Concordance index
DCA: Decision curve analysis
KM: Kaplan–Meier
HCM: Hypertrophic cardiomyopathy
DCM: Dilated cardiomyopathy
