# Supplementary material for: Eight-lncRNA signature of cervical cancer were identified by integrating DNA methylation, copy number variation and transcriptome data
Source: J Transl Med. 2021 Feb 8;19:58. doi: 10.1186/s12967-021-02705-9 (PMC8045209; doi:10.1186/s12967-021-02705-9)
Supplement: Supplementary file 1 — Additional file 1: Table S1. Distribution of differentially expressed lncRNA and PCGs in three subtypes. [file 12967_2021_2705_MOESM1_ESM.docx]

**Table 1.** Distribution of differentially expressed lncRNA and PCGs in three subtypes.

| **Type** | **Cluster1** | **Cluster2** | **Cluster3** |
| --- | --- | --- | --- |
| PCG_Down | 912 | 171 | 346 |
| PCG_Up | 77 | 271 | 124 |
| PCG_All | 989 | 442 | 470 |
| Lnc_Down | 319 | 117 | 131 |
| Lnc_Up | 36 | 127 | 68 |
| Lnc_All | 355 | 244 | 199 |
