# Supplementary material for: Eight-lncRNA signature of cervical cancer were identified by integrating DNA methylation, copy number variation and transcriptome data
Source: J Transl Med. 2021 Feb 8;19:58. doi: 10.1186/s12967-021-02705-9 (PMC8045209; doi:10.1186/s12967-021-02705-9)
Supplement: Supplementary file 2 — Additional file 2: Table S2. CGPs and lncRNA in 26 modules. [file 12967_2021_2705_MOESM2_ESM.docx]

**Table 2. CGPs and lncRNA in 26 modules.**

| **Module** | **All** | **Lnc** | **PCG** | **p.value** | **FoldChange** |
| --- | --- | --- | --- | --- | --- |
| green | 255 | 142 | 113 | 2.13E-24 | 3.556 |
| pink | 138 | 102 | 36 | 2.87E-32 | 8.017 |
| brown | 499 | 125 | 374 | 0.728 | 0.946 |
| turquoise | 1775 | 412 | 1363 | 0.999 | 0.855 |
| blue | 741 | 52 | 689 | 1 | 0.214 |
| magenta | 136 | 63 | 73 | 2.43E-07 | 2.442 |
| yellow | 435 | 83 | 352 | 1.000 | 0.667 |
| purple | 117 | 28 | 89 | 0.738 | 0.890 |
| tan | 106 | 35 | 71 | 0.067 | 1.395 |
| black | 165 | 35 | 130 | 0.941 | 0.762 |
| midnightblue | 93 | 30 | 63 | 0.109 | 1.347 |
| darkgrey | 62 | 18 | 44 | 0.345 | 1.158 |
| cyan | 99 | 26 | 73 | 0.525 | 1.008 |
| lightgreen | 82 | 26 | 56 | 0.151 | 1.314 |
| royalblue | 78 | 17 | 61 | 0.842 | 0.789 |
| red | 186 | 35 | 151 | 0.993 | 0.656 |
| lightyellow | 81 | 22 | 59 | 0.457 | 1.055 |
| greenyellow | 111 | 28 | 83 | 0.621 | 0.955 |
| darkgreen | 69 | 25 | 44 | 0.040 | 1.608 |
| salmon | 100 | 29 | 71 | 0.288 | 1.156 |
| grey60 | 82 | 13 | 69 | 0.991 | 0.533 |
| darkturquoise | 63 | 13 | 50 | 0.874 | 0.736 |
| orange | 55 | 7 | 48 | 0.995 | 0.413 |
| lightcyan | 88 | 8 | 80 | 1.000 | 0.283 |
| darkred | 75 | 10 | 65 | 0.998 | 0.435 |
