# Supplementary material for: Eight-lncRNA signature of cervical cancer were identified by integrating DNA methylation, copy number variation and transcriptome data
Source: J Transl Med. 2021 Feb 8;19:58. doi: 10.1186/s12967-021-02705-9 (PMC8045209; doi:10.1186/s12967-021-02705-9)
Supplement: Supplementary file 3 — Additional file 3: Table S3. Prognostic information of 41 lncrnas with significant prognosis. [file 12967_2021_2705_MOESM3_ESM.docx]

**Table S3. Prognostic information of 41 lncrnas with significant prognosis.**

| **Gene** | **p.value** | **HR** | **Low 95%CI** | **High 95%CI** |
| --- | --- | --- | --- | --- |
| ENSG00000235927 | 0.002238 | 6.695714 | 1.978294 | 22.66225 |
| ENSG00000203865 | 0.001287 | 1.388772 | 1.137063 | 1.696201 |
| ENSG00000225855 | 0.000283 | 1.075474 | 1.034043 | 1.118564 |
| ENSG00000230630 | 0.001425 | 1.031832 | 1.012153 | 1.051894 |
| ENSG00000233355 | 0.008181 | 0.376517 | 0.182548 | 0.77659 |
| ENSG00000236008 | 0.000757 | 2.404969 | 1.443205 | 4.00766 |
| ENSG00000236502 | 0.005757 | 1.09295 | 1.026129 | 1.164123 |
| ENSG00000237751 | 0.001895 | 1.093047 | 1.033375 | 1.156165 |
| ENSG00000226913 | 0.002924 | 5.528566 | 1.792581 | 17.05086 |
| ENSG00000281392 | 0.00571 | 10.10429 | 1.959749 | 52.09681 |
| ENSG00000273125 | 0.000261 | 12.76854 | 3.253924 | 50.10433 |
| ENSG00000214145 | 0.003799 | 1.177443 | 1.054149 | 1.315158 |
| ENSG00000248668 | 0.000631 | 1.443085 | 1.169368 | 1.780871 |
| ENSG00000237187 | 0.007102 | 1.135708 | 1.035211 | 1.245961 |
| ENSG00000281881 | 2.29E-08 | 1.383887 | 1.234843 | 1.550921 |
| ENSG00000249306 | 0.000966 | 1.195388 | 1.075173 | 1.329045 |
| ENSG00000225791 | 0.006028 | 1.103238 | 1.028529 | 1.183373 |
| ENSG00000230910 | 0.003268 | 3.569962 | 1.528939 | 8.335602 |
| ENSG00000231889 | 0.006142 | 1.346819 | 1.08847 | 1.666487 |
| ENSG00000227954 | 0.006216 | 1.353507 | 1.089664 | 1.681237 |
| ENSG00000273313 | 3.91E-06 | 1.725293 | 1.368647 | 2.174876 |
| ENSG00000224729 | 0.000104 | 4.320885 | 2.063747 | 9.046673 |
| ENSG00000253490 | 0.003629 | 0.006585 | 0.000223 | 0.194305 |
| ENSG00000253301 | 0.000767 | 1.227827 | 1.08945 | 1.38378 |
| ENSG00000248801 | 0.007689 | 1.10393 | 1.026515 | 1.187183 |
| ENSG00000229847 | 0.009811 | 1.02555 | 1.006099 | 1.045376 |
| ENSG00000254872 | 0.000242 | 1.017013 | 1.007892 | 1.026216 |
| ENSG00000247416 | 0.000241 | 3.873038 | 1.879774 | 7.979907 |
| ENSG00000255248 | 0.005119 | 1.037714 | 1.011163 | 1.064962 |
| ENSG00000245105 | 0.000328 | 1.398551 | 1.164661 | 1.679411 |
| ENSG00000229373 | 8.95E-05 | 1.819648 | 1.348696 | 2.455054 |
| ENSG00000260898 | 0.009955 | 0.016005 | 0.00069 | 0.37138 |
| ENSG00000267278 | 5.20E-06 | 1.368832 | 1.195919 | 1.566745 |
| ENSG00000234899 | 0.007495 | 2.151409 | 1.227011 | 3.772224 |
| ENSG00000265096 | 0.0051 | 1.761312 | 1.185204 | 2.617457 |
| ENSG00000268655 | 0.003262 | 1.244219 | 1.075658 | 1.439194 |
| ENSG00000269959 | 0.000153 | 1.852057 | 1.346113 | 2.548163 |
| ENSG00000205181 | 0.000343 | 1.252043 | 1.107098 | 1.415964 |
| ENSG00000226496 | 0.003458 | 1.170265 | 1.053196 | 1.300347 |
| ENSG00000225783 | 0.003271 | 1.005313 | 1.001769 | 1.00887 |
| ENSG00000234493 | 0.000155 | 1.273508 | 1.123566 | 1.44346 |
