# Supplementary material for: Eight-lncRNA signature of cervical cancer were identified by integrating DNA methylation, copy number variation and transcriptome data
Source: J Transl Med. 2021 Feb 8;19:58. doi: 10.1186/s12967-021-02705-9 (PMC8045209; doi:10.1186/s12967-021-02705-9)
Supplement: Supplementary file 4 — Additional file 4: Table S4. Information of 8-lncRNA signature. [file 12967_2021_2705_MOESM4_ESM.docx]

**Table S4. Information of 8-lncRNA signature.**

| **ENSG** | **coef** | **HR** | **Z-score** | **P value** | **Low 95%CI** | **High 95%CI** |
| --- | --- | --- | --- | --- | --- | --- |
| ENSG00000225855 | 0.486 | 1.625 | 2.638 | 0.008 | 1.133 | 2.332 |
| ENSG00000273125 | 2.707 | 14.991 | 3.500 | 0.0005 | 3.291 | 68.280 |
| ENSG00000249306 | 0.573 | 1.773 | 2.793 | 0.005 | 1.186 | 2.651 |
| ENSG00000253490 | -2.601 | 0.074 | -1.735 | 0.083 | 0.004 | 1.401 |
| ENSG00000130600 | 0.096 | 1.101 | 1.958 | 0.050 | 1.000 | 1.213 |
| ENSG00000229373 | 0.768 | 2.156 | 2.123 | 0.034 | 1.061 | 4.382 |
| ENSG00000260898 | -3.111 | 0.045 | -1.993 | 0.046 | 0.002 | 0.950 |
| ENSG00000265096 | 0.684 | 1.983 | 1.953 | 0.051 | 0.997 | 3.941 |
