# Supplementary figures and images for: Eight-lncRNA signature of cervical cancer were identified by integrating DNA methylation, copy number variation and transcriptome data
Source: J Transl Med. 2021 Feb 8;19:58. doi: 10.1186/s12967-021-02705-9 (PMC8045209; doi:10.1186/s12967-021-02705-9)

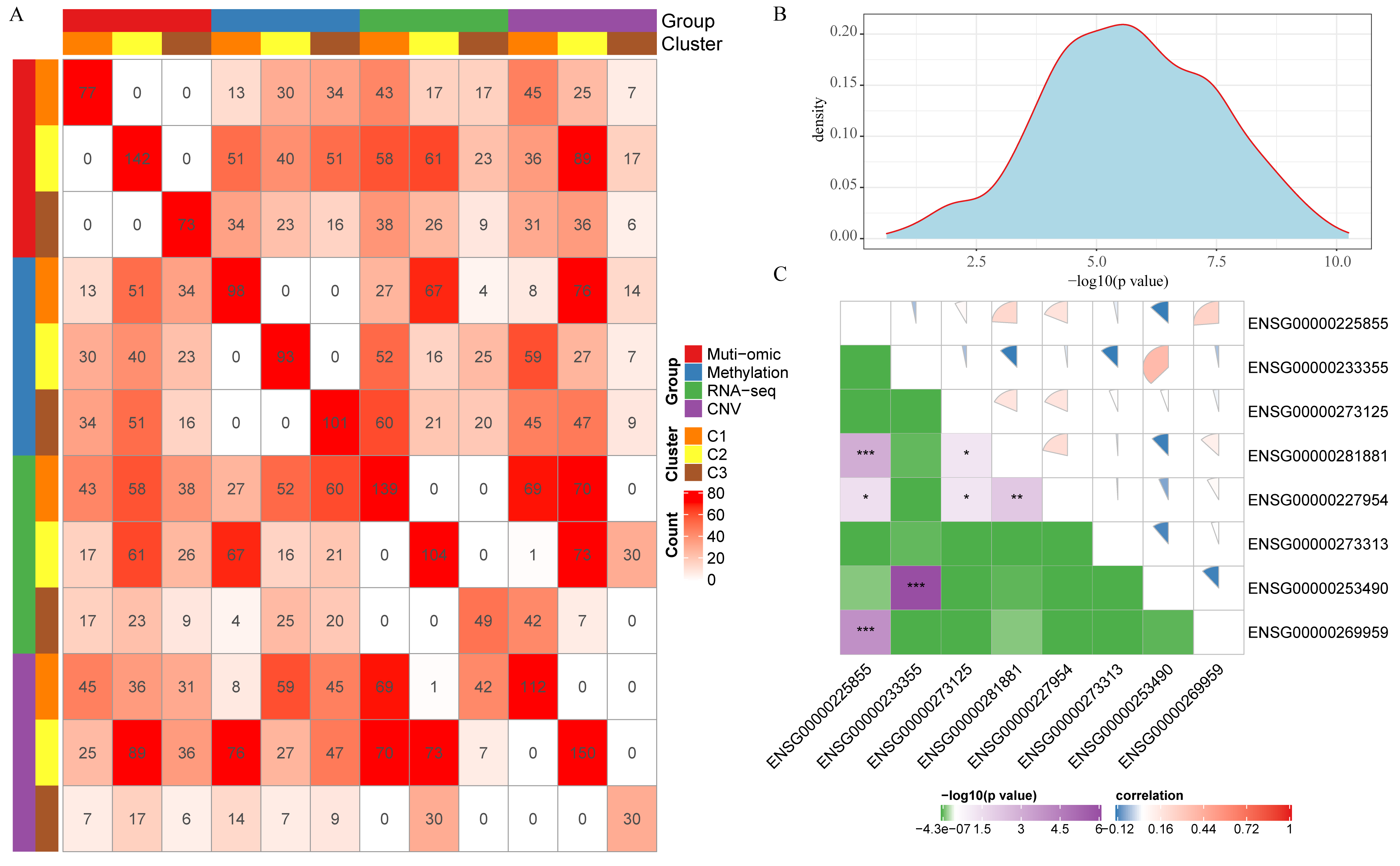

Supplement: Supplementary file 5 — Additional file 5: Figure S1. Advantage of multi-omics. a The results of multi-omics clustering were compared with those obtained by separate hierarchical clustering. b: Model was applied to the prognostic prediction of these one thousand samples, and the prognostic significance p-values were calculated for each calculation. c The correlation between 8 lncRNAs were analyzed. [file 12967_2021_2705_MOESM5_ESM.tif]

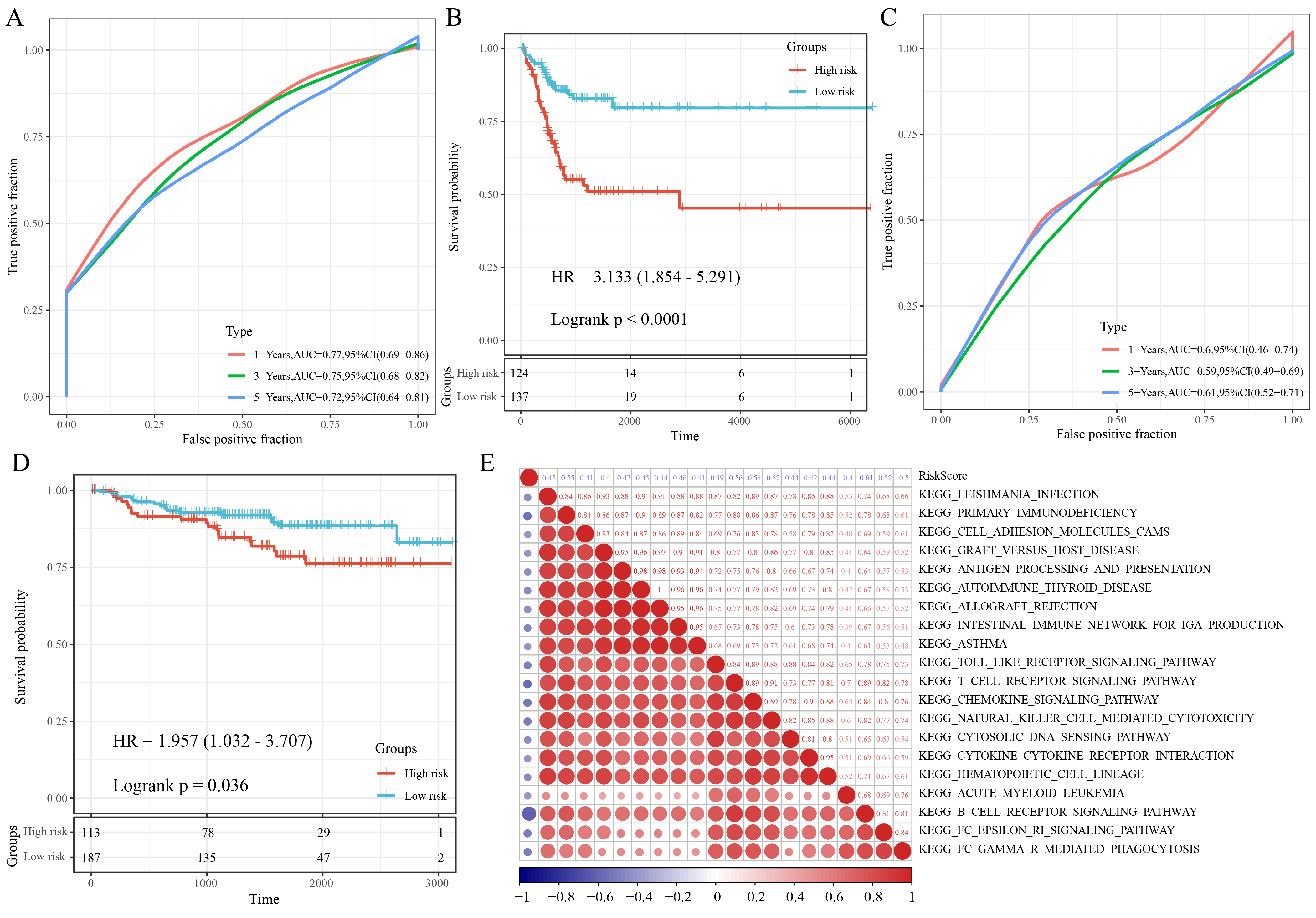

Supplement: Supplementary file 6 — Additional file 6: Figure S2. Prognostic model validation and functional analysis of the 8-lncRNA model. a ROC curve of the 8-lncRNA model in all TCGA datasets. Abscissa means false positive fraction, ordinate means true positive fraction. b KM survival curve distribution of the 8-lncRNA model in the high- and low-risk groups in all TCGA datasets. Abscissa means time, ordinate means survival probability. c ROC curve of the 8-lncRNA model in GSE44001 dataset. Abscissa means false positive fraction, ordinate means true positive fraction. d KM survival curve distribution of the 8-lncRNAs in the high- and low- risk group in GSE44001 datasets. Abscissa means time, ordinate means survival probability. e KEGG Pathway was the most correlated with the 8-lncRNA model, and the circle size in the figure indicates the correlation. [file 12967_2021_2705_MOESM6_ESM.tif]

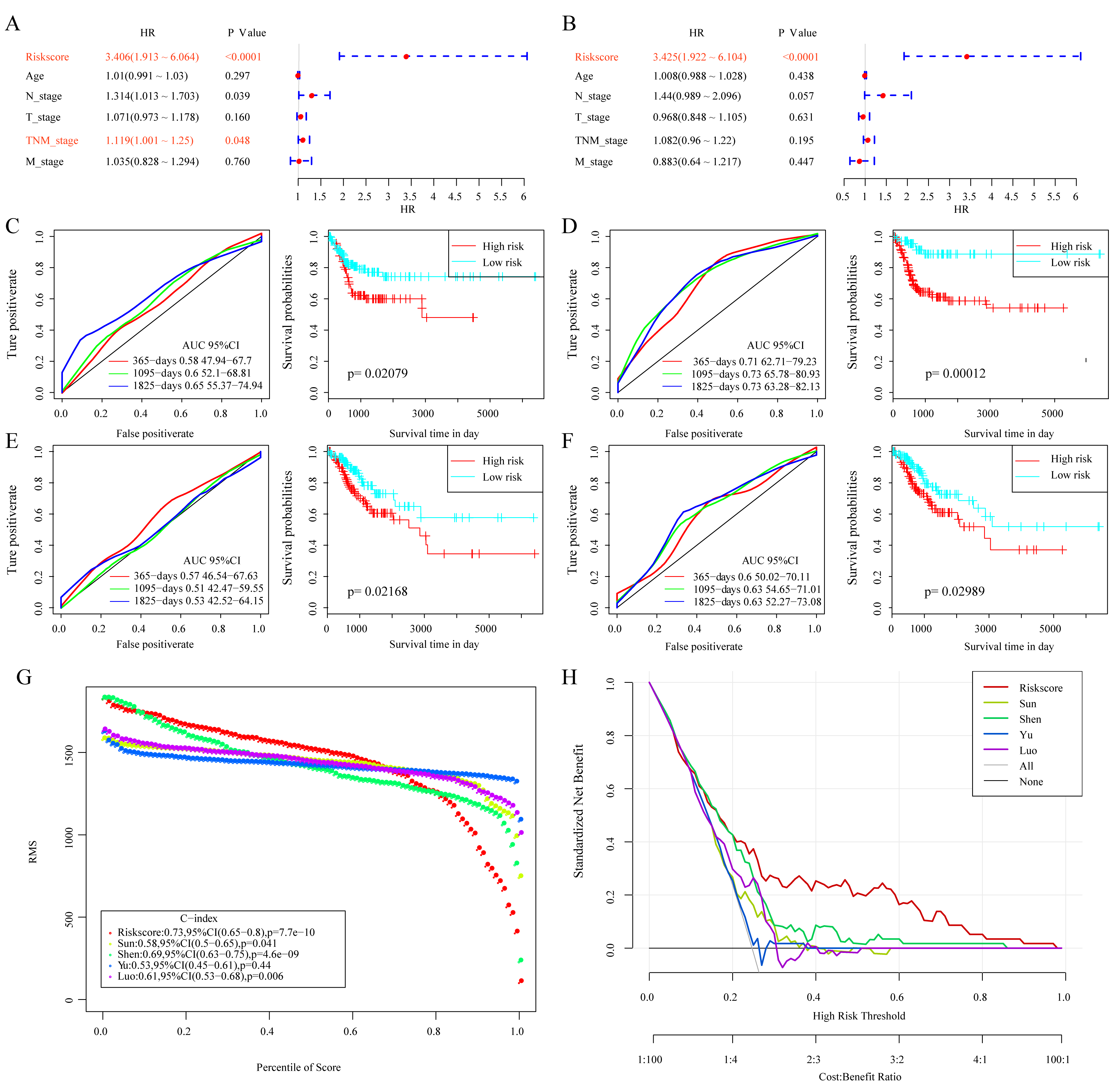

Supplement: Supplementary file 7 — Additional file 7: Figure S3. Comparison of the 8-lncRNA prognosis model with clinical features and the existing models a Forest characteristics of clinical features and risk score using univariate survival analysis. b Forest characteristics of clinical characteristics and risk score using multivariate survival analysis, and among them, orange-red represents a significant prognostic correlation. c ROC curve and KM curve of a 4-lncRNA signature in TCGA dataset. d ROC curve and KM curve of a 10-lncRNA signature in TCGA dataset. e ROC curve and KM curve of a 9-lncRNA signature in TCGA dataset. f OC curve and KM curve of a 6-lncRNA signature in TCGA dataset. Left: Abscissa means false positive fraction, ordinate means true positive fraction. Right: Abscissa means time, ordinate means survival probality. G: Comparison of restricted mean survival of five prognostic risk models. Abscissa means restricted mean survival, ordinate means percentiles of marker. h Comparison of decision curve analysis of the five prognostic risk models. Abscissa means threshold probability, ordinate means net benefit. [file 12967_2021_2705_MOESM7_ESM.tif]
